# Comparative genomics of 26 complete circular genomes of 18 different serotypes of *Actinobacillus pleuropneumoniae*

**DOI:** 10.1099/mgen.0.000776

**Published:** 2022-02-23

**Authors:** Valentina Donà, Alban Ramette, Vincent Perreten

**Affiliations:** ^1^​ Institute of Veterinary Bacteriology, Vetsuisse Faculty, University of Bern, Bern, Switzerland; ^2^​ Institute for Infectious Diseases, University of Bern, Bern, Switzerland

**Keywords:** porcine pleuropneumonia, WGS, ONT, serotype, LPS, CPS

## Abstract

*
Actinobacillus pleuropneumoniae* is a Gram-negative, rod-shaped bacterium of the family *Pasteurellaceae* causing pig pleuropneumonia associated with great economic losses worldwide. Nineteen serotypes with distinctive lipopolysaccharide (LPS) and capsular (CPS) compositions have been described so far, yet complete circular genomes are publicly available only for the reference strains of serotypes 1, 4 and 5b, and for field strains of serotypes 1, 3, 7 and 8. We aimed to complete this picture by sequencing the reference strains of 17 different serotypes with the MinION sequencer (Oxford Nanopore Technologies, ONT) and on an Illumina HiSeq (Illumina) platform. We also included two field isolates of serotypes 2 and 3 that were PacBio- and MinION-sequenced, respectively. Genome assemblies were performed following two different strategies, i.e. PacBio- or ONT-only *de novo* assemblies polished with Illumina reads or a hybrid assembly by directly combining ONT and Illumina reads. Both methods proved successful in obtaining accurate circular genomes with comparable qualities. blast-based genome comparisons and core-genome phylogeny based on core genes, SNP typing and multi-locus sequence typing (cgMLST) of the 26 circular genomes indicated well-conserved genomes across the 18 different serotypes, differing mainly in phage insertions, and CPS, LPS and RTX-toxin clusters, which, consistently, encode serotype-specific antigens. We also identified small antibiotic resistance plasmids, and complete subtype I-F and subtype II-C CRISPR-Cas systems. Of note, highly similar clusters encoding all those serotype-specific traits were also found in other pathogenic and commensal *Actinobacillus* species. Taken together with the presence of transposable elements surrounding these loci, we speculate a dynamic intra- and interspecies exchange of such virulence-related factors by horizontal gene transfer. In conclusion, our comprehensive genomics analysis provides useful information for diagnostic test and vaccine development, but also for whole-genome-based epidemiological studies, as well as for the surveillance of the evolution of antibiotic resistance and virulence genes in *A. pleuropneumoniae *.

## Data Summary

The complete genome and plasmid sequences of all strains sequenced in this study are deposited in DDBJ/ENA/GenBank under the accession numbers shown in Table 1.

**Table 1.** Strains and genome sequences used in this study

Strain	Serotype	Genome length (bp)	Coding sequences (total)	Accession no.	Reference
**S4074**	Serotype 1	2 318 657	2134	CP030753	[31]
**S1536**	Serotype 2	2 282 693	2095	CP031875	This study
**S1421**	Serotype 3	2 235 635	2029	CP031874	This study
**M62**	Serotype 4	2 335 268	2174	CP031873 (LS483358)*	This study
**K17**	Serotype 5a	2 284 762	2097	CP069797	This study
**L20**	Serotype 5b	2 274 482	2012	CP000569	[36]
**femø**	Serotype 6	2 409 565	2245	CP069796	This study
**WF83**	Serotype 7	2 312 414	2129	CP031869	This study
**405** **pAP8_1 (plasmid)** **pAP8_2 (plasmid**)	Serotype 8	2 311 859 5470 4063	2149 6 4	CP031866 CP031867 CP031868	This study
**CVJ13261**	Serotype 9	2 324 821	2144	CP031865	This study
**D13039**	Serotype 10	2 310 450	2123	CP031864	This study
**56153**	Serotype 11	2 324 505	2150	CP031863	This study
**8329**	Serotype 12	2 252 295	2059	CP031862	This study
**N273**	Serotype 13	2 308 478	2214	CP031861	This study
**3906**	Serotype 14	2 223 843	2033	CP031860	This study
**HS143**	Serotype 15	2 240 110	2039	CP031859	This study
**A-85/14**	Serotype 16	2 364 304	2192	CP069795	This study
**16287–1** **pAP17_1 (plasmid**)	Serotype 17	2 297 880 2310	2125 3	CP031856 CP031857	This study
**7311555**	Serotype 18	2 214 657	2019	CP031855	This study
**7213384-1**	Serotype 19	n/a	n/a	MT468887-9	[6]
KL16 Plasmid unnamed	Serotype 1	2 357 806 7699	2168 10	CP022715- CP022716	
HK361	Serotype 2	2 325 526	2144	LR134515	
P1875	Serotype 2	2 309 071	2092	CP079921	This study
JL03	Serotype 3	2 242 062	2036	CP000687	[37]
ORG1224	Serotype 3	2 245 829	2041	CP031854	This study
AP76 pAPP7_A (plasmid) pAPP7_B (plasmid) pAPP7_C (plasmid)	Serotype 7	2 331 981 5685 4236 3533	2131 5 4 2	CP001091 CP001093 CP001094 CP001092	
MIDG2331	Serotype 8	2 337 633	2106	LN908249	[38]
*A. porcitonsillarum* 9953 L55	n/a	2 263 191	2087	CP029206	[31]
* A. suis* NCTC12996	n/a	2 501 959	2263	LT906456	

Reference strains are highlighted in bold type.

*PacBio-sequenced M62 used for the comparison.

The data and software used to support the findings of this study are included within the article and the supplementary materials. Additional sequencing data are available from the corresponding author upon request.

**Impact Statement**

Herein, we performed comprehensive comparative genomics of 26 full genomes focusing on core-genome-inferred strain phylogeny and serotype-specific virulence traits, such as the lipopolysaccharide (LPS), capsular (CPS) and RTX-toxin antigenic regions, and highlighting the potential of a dynamic intra- and interspecies exchange of these factors by horizontal gene transfer. For this, we used seven genomes already available on NCBI and provided the complete circular genomes of 17 reference strains and two field isolates of *Actinobacillus pleuropneumoniae *, representing 18 of the 19 serotypes described so far. We used either long-read-only assemblies with Oxford Nanopore Technologies (ONT) followed by polishing of the scaffolds with Illumina reads, or a hybrid assembly approach combining both ONT and Illumina reads. We show that both methods yield complete circular genomes with comparable assembly qualities, therefore representing good backup options if the method of choice fails. In conclusion, our work provides simple and affordable workflows to obtain 26 accurate, circular genomes of *A. pleuropneumoniae *. The in-depth genome analysis presented here may serve as a basis for the development of diagnostic tests and vaccines, as well as for the establishment of whole-genome-based surveillance and epidemiological studies.

## Introduction

*Actinobacillus pleuropneumoniae* is a Gram-negative, facultatively anaerobic, rod-shaped bacterium belonging to the family *Pasteurellaceae *, and is the causative agent of porcine pleuropneumonia, a disease associated with high economic burdens in the pig industry worldwide [1].

Nineteen different serotypes (1 to 19) and two biotypes (based on NAD-dependency for growth) of *A. pleuropneumoniae* have been described so far, showing different virulence potential, as well as geographical distribution [1–6]. For instance, serotypes 1 and 5 are common in North and South America, while serotype 2 is common in Europe [7, 8]. On the other hand, those serotypes are absent in the UK [9].

The main factors involved in serotype-specific virulence and immunogenicity of *A. pleuropneumoniae* are usually well conserved among isolates belonging to the same serotype [10, 11]. For instance, strains belonging to beta-NAD-independent biotype 1 are usually less virulent than those of biotype 2, with occasional exceptions also observed [12, 13]. Other common virulence and immunogenicity factors harboured by *A. pleuropneumoniae* strains are serotype-specific capsular polysaccharides (CPS), outer membrane lipopolysaccharides (LPS) and RTX toxins, in addition to other outer membrane proteins, iron-acquisition proteins and adhesins [10, 14–16].

Serotyping of *A. pleuropneumoniae* is classically determined by antibody-based tests, with distinction of the serotypes being based on the presence of different surface carbohydrates; namely, the capsular polysaccharides of the cell envelope mainly define the serotype, while the lipopolysaccharides on the outer membrane rather aid in the distinction of subtypes within the same serotype [2, 17]. The genes involved in the biosynthesis of the capsular polysaccharides and O-specific polysaccharides are clustered on specific CPS and LPS loci. Previous analyses of these clusters confirmed a high diversity of involved genes and their genetic arrangement among the different serotypes [18, 19]. However, laboratories have increasingly switched to molecular serotyping methods, which guarantee an increased accuracy and reproducibility compared to the classic antibody-based methods [5]. In this regard, genes of the LPS and CPS clusters, as well as genes encoding the RTX toxins, are often chosen as targets for nucleic amplification-based assays, such as multiplex PCR, in order to conveniently discriminate among serotypes [6, 19–21].

On a different note, RTX toxins are of great interest within the field of vaccine development due to their high immunogenicity, but some outer membrane proteins and carbohydrate structures of CPS and LPS are also used to increase vaccine efficiency and may represent potential candidates for glycoconjugate vaccines, respectively [22–24].

Nonetheless, the mechanisms and pathways involved in serotype diversity are still poorly understood, both at the molecular and at the evolutionary level. Only the relatively recent advent of next-generation sequencing (NGS) has offered new powerful tools to elucidate such mechanisms [25]. In particular, the development of third-generation sequencing methods offering increased read length, but also a reduction of sequencing time, costs and PCR amplification bias, which have contributed to their rapid recent spread in the scientific community [26].

While most popular sequencing methods, such as Illumina (Illumina), ION Torrent (Thermo Fisher Scientific) and PacBio (Pacific Biosciences) are synthesis-based technologies, the innovative Oxford Nanopore Technologies (ONT) sequencing approach relies on directly monitoring the alterations in the ionic current caused by individual, native DNA molecules progressing through nanopores [27]. Therefore, the main advantage of ONT compared to the other methods is the generation of particularly long and unbiased sequence reads, because neither amplification nor chemical reactions are necessary for sequencing [28]. This technology also provides several other benefits; for example, the MinION sequencing device (ONT) is small (10×3×2 cm), portable, rapid, cheap and connects directly to a laptop through a USB 3.0 interface; the library construction involves simplified methods; no amplification step is required; and data acquisition and analysis occur in real-time. On the other hand, this technology is still relatively error-prone, thus often requiring additional correction, typically with accurate short Illumina reads, to improve consensus accuracy [29].

We have successfully applied ONT technology coupled with short-read Illumina sequencing to obtain full circular sequences of bacterial chromosomes and plasmids, including *Actinobacillus* [30, 31]. We showed that our sequencing and assembly strategy, i.e. the generation of an ONT-only draft consensus sequence that is subsequently polished with high-accuracy Illumina reads, yielded the full circular chromosome sequence of the *A. pleuropneumoniae* serotype 1 reference strain S4074 with comparable results to a PacBio-generated sequence of the same strain [31]. Despite the availability of such new technologies, only 10 complete circular genomes of *A. pleuropneumoniae* strains were available in the NCBI database at the time of this writing, covering just the reference strains of serotypes 1, 4 and 5b, and some additional field isolates of serotypes 2, 3, 7 and 8.

Here, we apply our sequencing strategy to complete the genomes of the reference strains of 17 different serotypes and of two additional field isolates (of which one strain was sequenced with the PacBio technology). We also validate the use of an alternative assembler, i.e. the Unicycler, that directly provides hybrid assemblies combining ONT and Illumina reads. The obtained genome sequences are then used together with the full genomes already available at NCBI (*n*=7) to infer strain phylogeny, as well as for further comparative genomics analyses, focusing especially on serotype-specific genomic features, such as the CPS, LPS and RTX clusters, in order to provide useful information for the design of new molecular typing methods or vaccines. We also propose rapid workflows to generate and analyse whole-genome sequences of *A. pleuropneumoniae *, including strain phylogeny, that can be easily applied for future surveillance and epidemiological studies.

## Methods

### Bacterial strains and growth conditions

All *A. pleuropneumoniae* strains belonging to serotypes 1–18 used in this study (Table 1) were grown overnight on chocolate agar plates supplemented with PolyViteX (bioMérieux) at 37 °C with 5 % CO_2_.

### DNA isolation and sequencing

DNA was isolated with a modified phenol/chloroform extraction method, treated for 30 min with 0.5 µl RNase (20 mg ml^−1^) (Qiagen) and purified with 0.8× Agencourt Ampure beads (Beckman-Coulter) [32]. The purified genomic DNA (gDNA) of all strains (except P1875) was subsequently used for library preparation for sequencing on the MinION sequencer (ONT), as described previously [31]. Briefly, the gDNA was sheared to 8–10 kb fragments with g-TUBES (Covaris) and library preparation was performed with the SQK-LSK108 1D ligation sequencing kit (ONT), as per the manufacturer’s instructions. The sequencing library was sequenced on a R9.4 SpotON flow cell (ONT) with the MinION Mk 1B sequencing device (ONT) for 24 h. Strain P1875 was sequenced separately with the PacBio (Pacific Biosciences) technology at the Functional Genomics Centre Zurich (FGCZ), Zurich, Switzerland. In parallel, gDNA of all strains was also submitted to Eurofins, Constance, Germany, for 2×150 paired-end sequencing on an Illumina HiSeq platform.

### Genome assembly

Base calling and quality filtering of ONT reads was performed with Albacore v2.0.1 (serotypes 1–4 and 7–18) and Guppy v3.1.5 (serotypes 5a and 6). Pairing, trimming and quality filtering of the Illumina reads was performed with trimmomatic v0.33. The genome assemblies were obtained with two different methods. The assemblies of all strains except the serotype 16 reference strain were obtained as described previously [31]. Briefly, PacBio and ONT reads were assembled with Canu v1.6 with default parameters and the option corOutCoverage=100. Paired-end Illumina reads were then mapped to the PacBio- or Canu-generated scaffold with BWA-MEM v0.7.13 and polished twice with Pilon 1.22.

For the serotype 4 and 16 reference strains, both ONT and Illumina reads were directly used for assembling with the Unicycler assembler [33, 34]. The final circular genome sequences were first all annotated with Prokka v1.12 for primary sequence analysis and subsequently with the NCBI prokaryotic genome annotation pipeline during the submission process. Paired-end Illumina reads were used to run plasmidSPAdes v3.9.0 with default parameters to assess the presence of small plasmids [35].

### Genome analysis and comparison

The main features of the complete genome sequences obtained in this study are summarized in Table 1. Additionally, we also retrieved for the present genome comparisons the PacBio-sequenced genomes of the reference strains S4074 (serotype 1), M62 (serotype 4) and L20 (serotype 5b), as well as the field isolates KL16 (serotype 1), HK361 (serotype 2), JL03 (serotype 3) and MIDG2331 (serotype 8), which were already deposited in the NCBI database (Table 1) [31, 36–38].

Phylogenetic analysis of the core genome was performed with SeqSphere v1.0 (Ridom), Roary v3.11.2 and Panseq v3.1.0 with default parameters [39, 40]. Visualization of the gene distribution of the pangenome was performed with Phandango v1.3.0 [41]. The CPS and LPS loci were identified by similarity searches of common genes belonging to the CPS and LPS clusters (e.g. *cpxD* for CPS; *rmlBADC* and *wbaP* for LPS). The complete CPS and LPS loci, which were found in all *Actinobacillus* species between *modF* and *ydeN*, and between *erpA* and *rpsU*, respectively, were extracted for phylogenetic analysis as previously done [19]. Phylogenetic analysis of the CPS and LPS regions was performed with the Geneious Prime software 2021.1 (https://www.geneious.com) by first obtaining nucleotide sequence alignments with muscle v3.8.425, which were subsequently used for the generation of trees with FastTree v2.1.11 using Generalized Time-Reversible (GTR) and optimizing the Gamma 20 likelihood. The CPS (accession number MT468887) and LPS (accession number MT468889) sequences of the recently described serotype 19 were included in the analysis [6].

Further genomics analyses were performed as described previously [31]. Briefly, genome alignments were performed with progressiveMauve v2.3.1 and LASTZ v1.02.00 present on the Geneious Prime software v2021.1 (https://www.geneious.com), and online available platforms were used to characterize the presence of orthologous gene clusters (Orthovenn), known resistance genes (ResFinder), plasmids (PlasmidFinder), insertion sequences (IS) (ISfinder), clustered regularly interspaced short palindromic repeat arrays and CRISPR-associated genes (CRISPR/Cas) systems (CRISPRone), and phage sequences (PHASTER). Circular maps obtained by blast-based comparisons of the 26 genome sequences were generated with the blast ring image generator (BRIG).

### Nucleotide sequence GenBank accession numbers

The complete nucleotide sequences of *A. pleuropneumoniae* reference strains obtained in this study were deposited in DDBJ/EMBL/GenBank under the accession numbers shown in Table 1.

## Results and discussion

### Assembly of the 19 *A. pleuropneumoniae* genome sequences

After base calling and quality filtering of the sequence reads, the complete circularized genome sequences of all strains except serotype 16 reference strain A-85/14 were obtained as described previously [31], assembling the ONT reads first to obtain a genome scaffold with the Canu assembler, followed by subsequent polishing with paired-end Illumina reads. However, for strain A-85/14, Canu assemblies consistently generated a single contig without overlapping ends, even after *ex novo* resequencing and tuning assembly parameters (data not shown). Only a hybrid assembly with reads from ONT and Illumina using the recently released Unicycler assembler finally provided a single, circular genome sequence [33]. Of note, this assembler directly combines ONT and Illumina reads, including a final polishing step with the Illumina reads, and therefore requires fewer file manipulations.

To validate the validity of both assembly strategies for *Actinobacillus* genomes, we additionally sequenced and assembled the genome of the serotype 4 reference strain M62 with the Unicycler assembler, in order to subsequently compare our Canu- and Unicycler-generated assemblies with the PacBio-sequenced complete genome of the same strain, which we retrieved from the NCBI genome database (accesssion number LS483358). Alignment of the three genomes indicated high structural homology and sequence identity (data not shown). The main difference in the Unicycler assembly compared to the other two assemblies was that the Unicycler assembly was missing one copy of a tRNA-Val quintuplet present in the other two assemblies. This finding was not surprising, since the assembly strategy of the Unicycler is based on short read (Illumina)-based contigs generated with SPAdes that are subsequently bridged together implementing the long reads. Therefore, known shortcomings of SPAdes-based assemblies, such as a lower resolution of (short) repetitive regions such as multiple t-RNA copies, should be expected. On the other hand, we were able to obtain the circular genome scaffold of the serotype 5a reference strain using the Canu assembler alone, since the Unicycler was not able to resolve a large rearrangement between two *rrn* operon regions present only in this strain (Fig. S1, available in the online version of this article).

### General features and strain phylogeny of the 26 *A. pleuropneumoniae* genomes

As shown in Table 1, the circular genomes of the 19 reference strains and the seven additional isolates ranged from 2 214 657 to 2 409 565 bp in length, containing an average GC content of 41.2 %, and between 2019 and 2245 coding sequences (CDS).

blast-based genome comparisons of the 19 reference strains and the seven additional field isolates confirmed a high sequence identity and structural homology among all genomes, which differed mainly in the presence or absence of intact and/or partial prophage sequences (Figs S1 and S2). Recombination at phage insertion sites or other large repetitive regions such as *rrn* operons were probably responsible for the few large inversions detected in the genomes of some strains, such as the serotype 5a reference strain K17, the serotype 16 reference strain A-85/14 and the serotype 2 field isolate P1875 (Figs S1 and S2).

Such findings were confirmed by further analysis of the pangenome performed with Roary (Figs S3 and S4). The pangenome of the 26 strains consisted of 4116 genes, of which there were: 1494 cloud genes (contained in <15 % of the strains); 861 shell genes (contained in ≥15 to <95 % of the strains); 94 soft core genes (contained in ≥95 to <99 % of the strains); and 1667 core genes (contained in >99 % of the strains) (Table S1). Overall, the core genome determined from the 26 genome sequences using Roary and SeqSphere contained 1667 and 1562 genes, respectively. The lower number observed with the latter was mainly attributed to the different filtering parameters, i.e.; that is, SeqSphere discards paralogues rather than splitting them into separate clusters, such as with Roary. Intriguingly, analysis with Orthovenn2 indicated the presence of 1597 single-copy clusters of orthologues genes (COGs) shared between *A. pleuropneumoniae* and the reference strain 9953 L55 of the non-pathogenic ‘*Actinobacillus porcitonsillarum*’ (accesssion number CP029206) [31]. Regarding species identification, average nucleotide identity (ANI) values among different *Actinobacillus* species including those of this paper were already computed by us and others in a previous study [31].

**Fig. 1.**
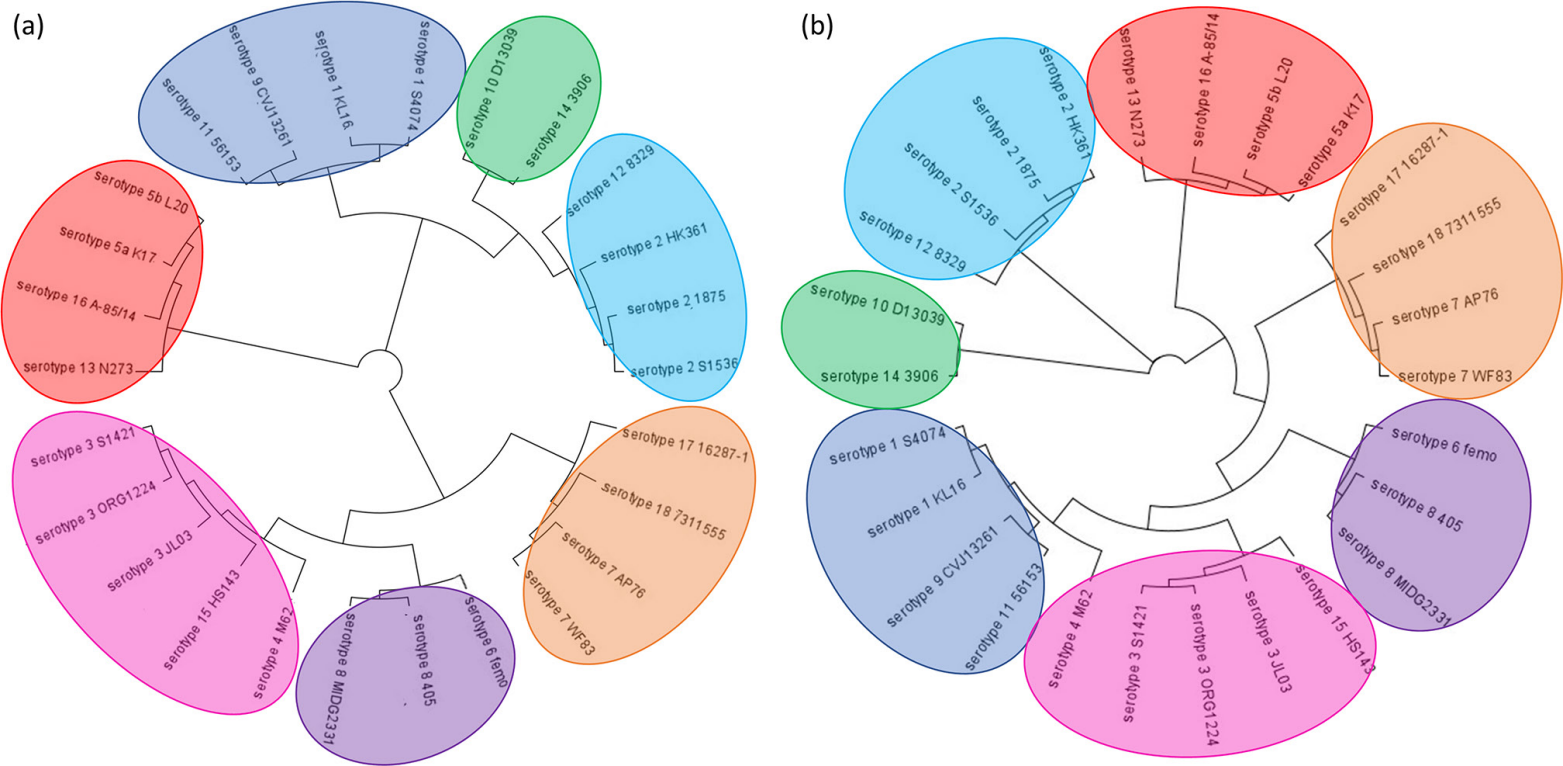
Phylogenetic analysis of the 26 *A *. *pleuropneumoniae* strains. The trees were generated with FastTree based on core-genome gene alignments performed with Roary (**a**) or core-genome SNP typing performed with Panseq (**b**). Groups of closely related serotypes are highlighted with different colours.

As shown in Fig. 1(a), analysis of the core-genome phylogeny of the 26 strains based on sequence alignments of the 1673 core genes identified by Roary shows clustering in seven groups of closely related serotypes: (i) serotypes 1, 4, 9 and 11; (ii) serotypes 2 and 12; (iii) serotypes 3 and 15; (iv) serotypes 5a, 5b, 13 and 16; (v) serotypes 6, 8 and 17; (vi) serotypes 7 and 18; and (vii) serotypes 10 and 14. These results were consistent with the phylogeny inferred by core-genome SNP typing and core-genome multi-locus sequence typing (cgMLST) obtained with Panseq and SeqSphere, respectively (Figs 1b and S5). As expected, serotype-specific genes comprised the CPS, LPS and RTX-toxin clusters, partially reflecting the grouping observed in the core-genome phylogeny.

### CPS and LPS clusters

As already described previously, the CPS locus in all *A. pleuropneumoniae* strains was located between the *modF* and *ydeN* genes and consisted of a first region harbouring the highly conserved export genes *cpxABCD* contiguous to a variable second region containing the biosynthetic *cps* gene cluster, while a third region encoding proteins for post-polymerization modification/transport was found elsewhere in the chromosome [18, 19]. The *cpxABCD* export operon and the biosynthetic *cps* genes were pointing in opposite directions in all 19 serotypes except for the serotype 15 reference strain, in which all genes were unidirectional [18, 19].

Interestingly, the frameshift splitting the *cpsD* gene in the serotype 3 isolate JL03 was not present in both the serotype 3 reference strain or the clinical isolate ORG1224, suggesting that this *cpsD* alteration is a strain-specific characteristic [18, 19]. Similarly, the serotype 7 reference strain WF83 did not harbour the IS *Apl1* located immediately upstream of the *cpsABCD* operon that is found in the serotype 7 AP76 isolate. Of note, this particular IS, typically found in *Actinobacillus* species, owes its popularity to its role in the worldwide spread of the colistin-resistance gene *mcr-1* in *Enterobacterales* and other bacterial species [30, 31].

**Fig. 2.**
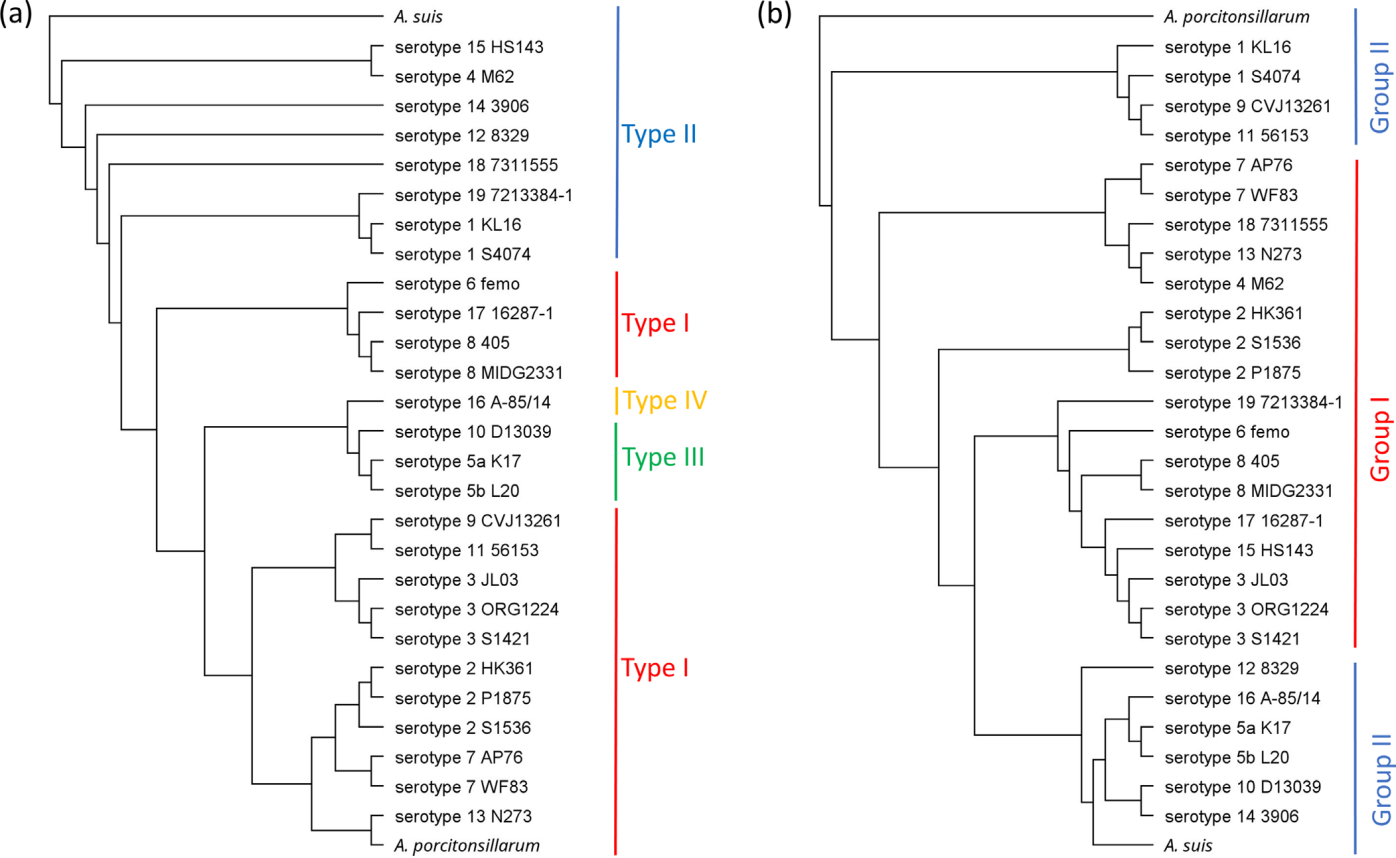
Phylogenetic analysis of the CPS (**a**) and LPS (**b**) clusters of the 27 *A *. *pleuropneumoniae* strains. The trees were generated based on nucleotide sequence alignments of the full region. The *A. suis* reference strain NCTC12996 (**a**) and the *A. porcitonsillarum* reference strain 9953 L55 (**b**) were used as root, respectively.

Analysis of the CPS locus of the 26 strains together with the recently proposed serotype 19 sequence (accession number MT468887) confirmed the presence of four different types, as described previously [6, 19]. As shown in Fig. 2(a), most serotypes harboured a type I or type II CPS cluster. Interestingly, the type I CPS loci of the serotype 1 strains clustered together with the serotype 18, while serotypes 9 and 11 shared an identical type II CPS locus. The serotype 16 CPS locus was the sole type IV member, while only serotypes 5 and 10 possessing type III CPS clusters.

Of note, there are several indications that the *A. pleuropneumoniae* CPS locus was first acquired by horizontal gene transfer, i.e.: the region shows an around 10 % lower GC content and is flanked by inverted repeats (IRs) in all 19 serotypes, followed by diversification of the capsule biosynthesis genes due to additional gene acquisition, deletion and/or horizontal transfer [19, 42].

Other *Actinobacillus* species, such as *A. suis* and ‘*A. porcitonsillarum*’, harbour similar CPS loci, suggesting potential interspecies transfer [19, 31, 43]. We therefore included the genome sequence of the *A. suis* reference strain NCTC12996 (accession number LT906456) and ‘*A. porcitonsillarum*’ 9953 L55 (accession number CP029206) for further analysis. Based on the phylogenetic relationship estimated by sequence alignments of the full CPS loci, the ‘*A. porcitonsillarum*’ and *A. suis* loci clustered together with the *A. pleuropneumoniae* type I and type II CPS clusters, respectively. Therefore, the diversity of serotypes found in pathogenic species probably reflects the evolutionary advantage of acquiring complete CPS clusters as a disguise mechanism to escape host immunity.

While the CPS locus mainly defines the serotype, subgrouping is based on the LPS cluster. Interestingly, the LPS cluster of all 19 serotypes of *A. pleuropneumoniae* [i.e. including the LPS region (accession number MT468889) of the recently proposed serotype 19] was located between the *erpA* and *rpsU* genes and resembled those found in the commensal ‘*A. porcitonsillarum*’ [6, 31], again suggesting a potential interspecies acquisition through horizontal gene transfer from non-pathogenic actinobacilli (Fig. 2a, b).

Evidence in this direction is also given when comparing the phylogenetic relationship of the CPS and LPS clusters of serotypes 1, 9 and 11. In contrast to the CPS locus, phylogenetic analysis of the LPS locus shows that serotypes 1, 9 and 11 cluster together (Fig. 2a, b). While the CPS clusters of the serotype 9 and the serotype 11 reference strains are identical, the LPS clusters only differ by two non-synonymous mutations, i.e. one in *rmlC*, encoding a subunit of the ABC-transporter, and another in a gene probably encoding a mannosyltransferase. Therefore, these mutations probably play an important role in the distinction between serotypes 9 and 11 by serotyping. Regardless, our data suggest a common ancestor for serotypes 1, 9 and 11, followed by the diversification into different serotypes due to the acquisition of different CPS loci. Considering that the LPS loci of these three serotypes and ‘*A. porcitonsillarum*’ cluster together (Fig. 2b), it is tempting to speculate that serotype 1 originated only eventually after the acquisition of a type II CPS cluster from another *Actinobacillus* species, such as *A. suis* (Fig. 2a). Thereafter, the acquisition of the non-synonymous mutations in the LPS cluster were responsible for the differentiation between serotypes 9 and 11.

There is more evidence pointing towards dynamic intra- or interspecies exchanges of these loci, such as the clustering of the group II LPS loci into two separate groups, i.e. either resembling the *A. suis* or the ‘*A. porcitonsillarum*’ LPS clusters (Fig. 2b), as well as the grouping of serotype 5 with serotype 16 in all phylogenetic analyses (Fig. 1), despite possessing different CPS types (Fig. 2a).

### RTX-toxin clusters

While not all virulence factors responsible for the development and severity of pneumonic lesions in pigs have yet been well established, the expression of particular exotoxins is known to influence the virulence of *A. pleuropneumoniae* [15, 44]. In particular, the RTX toxins ApxI, ApxII and ApxIII, which can be found in serotype-dependent combinations, are responsible for its haemolytic phenotype and CAMP positivity [15, 45].

RTX-toxin expression depends on *apxCABD* operons, with the genes *apxCA* and *apxBD* responsible for the production and secretion of the toxin, respectively [15, 46]. All serotypes except serotypes 10 and 14 harboured the partial *apxIICA* operon for ApxII biosynthesis (Table 2). Since this operon lacks the export genes, toxin secretion in these strains probably depends on the secretion system of either ApxI or ApxIII. In fact, all serotypes containing the *apxIIICABD* operon except the serotype 3 strains also harbour a partial *apxIBD* operon encoding the type I secretion system ATPase and its periplasmic adaptor subunit, which makes the ApxI secretion system probably responsible for ApxII secretion. A serotype 3 variant was found lacking also the partial *apxII* operon, while the closely related serotype 15 still harbours a partial *apxIBD* operon [47]. Therefore, the production of ApxI and ApxII toxins in serotype 3 strains may not be advantageous.

**Table 2.** Genes encoding the RTX toxins in the 27 *A *. *pleuropneumoniae* strains

Strain	Serotype	*apxI* operon	*apxII* operon	*apxIII* operon
**S4074**	Serotype 1	*apxIC apxIA apxIB apxID*	*apxIIC apxIIA*	
**S1536**	Serotype 2	*apxIB apxID*	*apxIIC apxIIA*	*apxIIIC apxIIIA apxIIIB apxIIID*
**S1421**	Serotype 3		*apxIIC apxIIA*	*apxIIIC apxIIIA apxIIIB apxIIID*
**M62**	Serotype 4	*apxIB apxID*	*apxIIC apxIIA*	*apxIIIC apxIIIA apxIIIB apxIIID*
**K17**	Serotype 5a	*apxIC apxIA apxIB apxID*	*apxIIC apxIIA*	
**L20**	Serotype 5b	*apxIC apxIA apxIB apxID*	*apxIIC apxIIA*	
**femø**	Serotype 6	*apxIB apxID*	*apxIIC apxIIA*	*apxIIIC apxIIIA apxIIIB apxIIID*
**WF83**	Serotype 7	*apxIB apxID*	*apxIIC apxIIA*	
**405**	Serotype 8	*apxIB apxID*	*apxIIC apxIIA*	*apxIIIC apxIIIA apxIIIB apxIIID*
**CVJ13261**	Serotype 9	*apxIC apxIA apxIB apxID*	*apxIIC apxIIA*	
**D13039**	Serotype 10	*apxIC apxIA apxIB apxID*		
**56153**	Serotype 11	*apxIC apxIA apxIB apxID*	*apxIIC apxIIA*	
**8329**	Serotype 12	*apxIB apxID*	*apxIIC apxIIA*	
**N273**	Serotype 13	*apxIB apxID*	*apxIIC apxIIA*	
**3906**	Serotype 14	*apxIC apxIA apxIB apxID*		
**HS143**	Serotype 15	*apxIB apxID*	*apxIIC apxIIA*	*apxIIIC apxIIIA apxIIIB apxIIID*
**A-85/14**	Serotype 16	*apxIC apxIA apxIB apxID*	*apxIIC apxIIA*	
**16287-1**	Serotype 17	*apxIB apxID*	*apxIIC apxIIA*	
7311555	Serotype 18	*apxIC apxIA apxIB apxID*	*apxIIC apxIIA*	
**7213384-1**	Serotype 19	*apxIB apxID*	*apxIIC apxIIA*	
KL16	Serotype 1	*apxIC apxIA apxIB apxID*	*apxIIC apxIIA*	
HK361	Serotype 2	*apxIB apxID*	*apxIIC apxIIA*	*apxIIIC apxIIIA apxIIIB apxIIID*
P1875	Serotype 2	*apxIB apxID*	*apxIIC apxIIA*	*apxIIIC apxIIIA apxIIIB apxIIID*
JL03	Serotype 3		*apxIIC apxIIA*	*apxIIIC apxIIIA apxIIIB apxIIID*
ORG1224	Serotype 3		*apxIIC apxIIA*	*apxIIIC apxIIIA apxIIIB apxIIID*
AP76	Serotype 7	*apxIB apxID*	*apxIIC apxIIA*	
MIDG2331	Serotype 8	*apxIB apxID*	*apxIIC apxIIA*	*apxIIIC apxIIIA apxIIIB apxIIID*

Reference strains are highlighted in bold type.

As already observed for the CPS and LPS clusters, similar operons for toxin expression are found also in other *Actinobacillus* species. For instance, intact *apxIICABD* operons are found in non-pathogenic species, such as ‘*A. porcitonsillarum*’ and *A. minor* [31]. Sequence alignments show that the *A. pleuropneumoniae* partial *apxIICA* operon is almost identical to the *apxIICA* part of the commensal complete operon (100 % coverage and 99.4 % identity), again suggesting interspecies transfer through mobile genetic elements. In fact, an IS*481* family transposase followed by an IS*595* family transposase are located immediately downstream the partial operon of *A. pleuropneumoniae* serotypes 1, 7, 17 and 18, or four genes downstream in all other serotypes.

Similarly, full *apxICABD* operons were found in *A. suis* by blastn search in the NCBI database, while the *apxIIICABD* cluster of *A. pleuropneumoniae* showed 98 % coverage and 82 % identity to the *apxCABD* operon of *Pasteurella aerogenes *. Previous studies have already pointed to the presence of Apx toxins and proteins in other *Actinobacillus* species and closely related members of the family *Pasteurellaceae *, which could immunologically cross-react with the Apx toxins of *A. pleuropneumoniae* and, therefore, interfere in the serodiagnosis of porcine pleuropneumonia [48–50]

### Plasmids and antimicrobial resistance genes

As for other Gram-negatives, plasmids play a major role as vectors for the exchange of resistance genes between the same or even different *Actinobacillus* species [51]. *In silico* analysis with PlasmidFinder and plasmidSPAdes using paired-end Illumina reads suggested that only serotype 8 and 16 reference strains (405 and A-85/14), as well as two clinical isolates (serotype 1 strain KL16, and serotype 7 strain AP76) contained small plasmids. Plasmids pAP8_1 and pAP8_2 carried by strain 405 harboured genes conferring resistance to tetracycline [*tet*(H)] and sulfonamide (*sul2*), respectively. The latter was identical to the *A. pleuropneumoniae* plasmid pARD3079, and highly similar (90 % query cover and 98.8 % identity) to the ‘*A. porcitonsillarum’* plasmid pKMA757 described previously, confirming that commensal species play a role as reservoirs of such small antimicrobial resistance plasmids [51].

Antimicrobial resistance genes were also found in plasmid unnamed1 of serotype 1 KL16 (*floR*; resistance to chloramphenicol/florfenicol), as well as in pAPP7_A (*bla*_ROB-1_; resistance to beta-lactams) and pAPP7_B (*strA* and *sul2* resistance to streptomycin/spectinomycin and sulfonamides, respectively) of serotype 7 AP76. Of note, only tetracycline resistance genes were identified when analysing the chromosomes of the 26 strains with Resfinder, i.e. *tet*(B) in the serotype 1 strain KL16, serotype 7 strain AP76 and serotype 8 strain MIDG2331, as well as *tet*(O) in the serotype 7 reference strain WF83. The tetracycline resistance operon containing *tet*(B) was located on a Tn*10* mobile element flanked by two IS*Vsa5* in opposite orientation, which is widely disseminated among different bacterial species and was also found on the chromosome and plasmids of commensal *Actinobacillus* species [31, 51].

### CRISPR-Cas systems

**Fig. 3.**
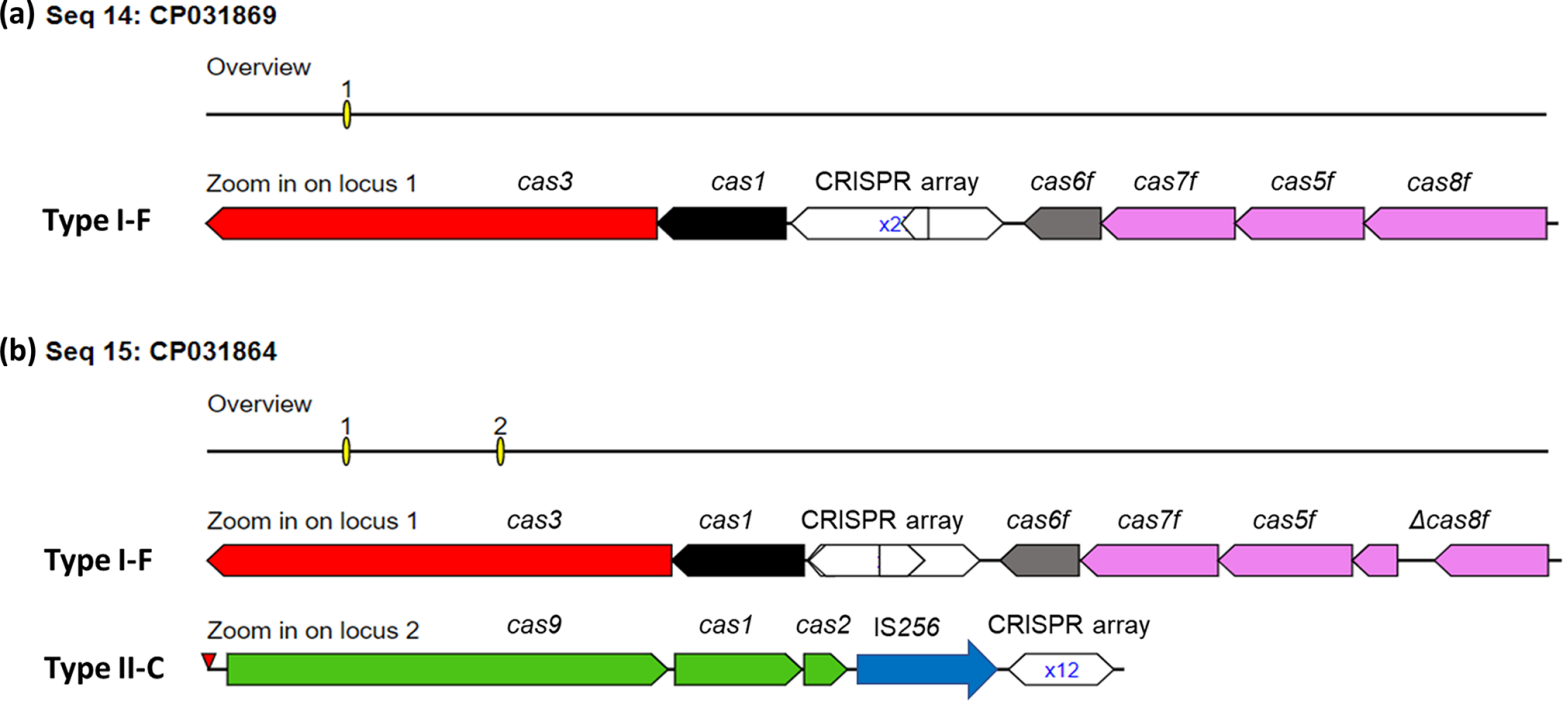
CRISPR-Cas systems of the 26 *A *. *pleuropneumoniae* strains. Schematic representation of the subtype I-F CRISPR-Cas system variant identified in all 26 *A *. *pleuropneumoniae* strains, differing only in the number of CRISPR repeats (**a**). The serotype 10 reference strain D13039 harboured a subtype I-F CRISPR-Cas system with a disrupted *cas8f* gene, as well as a complete type II-C CRISPR-Cas system with an IS*256* immediately upstream of the CRISPR array (**b**).

It has been recently shown that bacterial immunity against bacteriophages depends to some extent on the presence of different types of CRISPR/Cas systems [52–54]. Previous observations have already indicated the presence of such defence systems also in *Actinobacillus* species [31]. Analysis of the 26 strains with CrisprONE confirmed that all *A. pleuropneumoniae* strains possessed a new variant of a complete subtype I-F CRISPR/Cas system with CRISPR arrays containing between 11 and 45 repeat units (Fig. 3a). Interestingly, only the serotype 10 reference strain D13039 harboured additionally a complete subtype II-C CRISPR/Cas system with a 12-unit CRISPR array (Fig. 3b). Since the *cas8* gene of the I-F CRISPR/Cas locus is disrupted, the type II-C system was eventually acquired in this specific strain to compensate for the faulty type I-F system.

The type II-C CRISPR/Cas system found in D1039 was highly similar to the one found previously in ‘*A. porcitonsillarum*’ and *A. minor* [31], as well as in the *A. suis* ATCC 33415 type strain (>96 % nucleotide identity by blast), with the exception of an IS*256* family transposase located immediately upstream of the CRISPR array. Similarly, an IS*Apl3* transposase is found immediately downstream of the type I-F CRISPR/Cas locus, which was almost identical to the one found in *A. lignieresii* strain NCTC10568 (accession number LR134169). Together, these observations again support the potential for interspecies exchange of different full CRISPR/Cas system clusters among *Actinobacillus* species.

## Conclusions

We have implemented both ONT and PacBio sequencing combined with Illumina to obtain 19 complete circular genome sequences of *A. pleuropneumoniae *, covering all 18 different serotypes known so far, and highlighted their main genetic features and differences.

Our data provide important insights into both general and serotype-specific genetic characteristics of *A. pleuropneumoniae *, such as core-genome phylogeny, antimicrobial resistance genes and CRISPR/Cas systems, as well as the analysis of the CPS, LPS and RTX-toxin clusters, which can be useful for future applications, such as the design of diagnostic tests, serotyping and vaccine development. In this regard, we also recommend considering the potential of a dynamic exchange of such virulence factors, surface antigens and resistance genes between the pathogen *A. pleuropneumoniae* and other pathogenic and non-pathogenic *Actinobacillus* species.

Finally, whole-genome sequencing was recently proposed for routine monitoring of the antimicrobial resistance profiles and the prompt identification of emerging resistances [55, 56]. In this context, the recent advances of whole-genome sequencing technologies and workflows, such as those presented in our work, support their implementation as the future standard for surveillance and epidemiological studies.

## Supplementary Data

Supplementary material 1

Supplementary material 2
