# Supplementary material for: Comparative genomics of 26 complete circular genomes of 18 different serotypes of Actinobacillus pleuropneumoniae
Source: Microb Genom. 2022 Feb 23;8(2):000776. doi: 10.1099/mgen.0.000776 (PMC8942016; doi:10.1099/mgen.0.000776)
Supplement: Supplementary material 1 [file mgen-8-0776-s001.pdf]

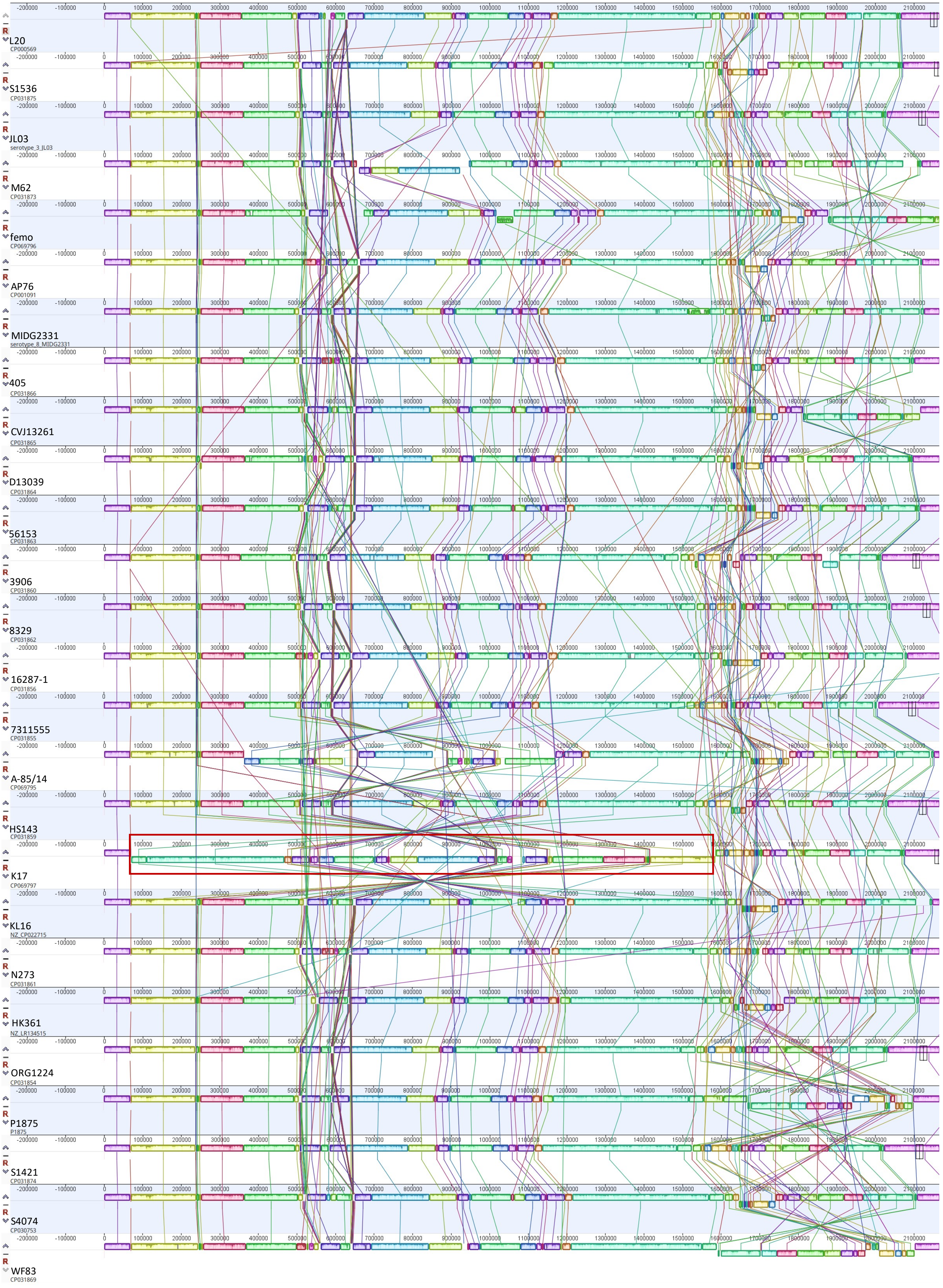

Figure S1. Whole-genome alignments of the 26 *A. pleuropneumoniae* strains generated with progressiveMAUVE. Same color boxes, i.e., locally collinear blocks (LCB), represent homologous regions of sequence without rearrangement. The red box highlights the big rearrangement found in the serotype 5a reference strain.

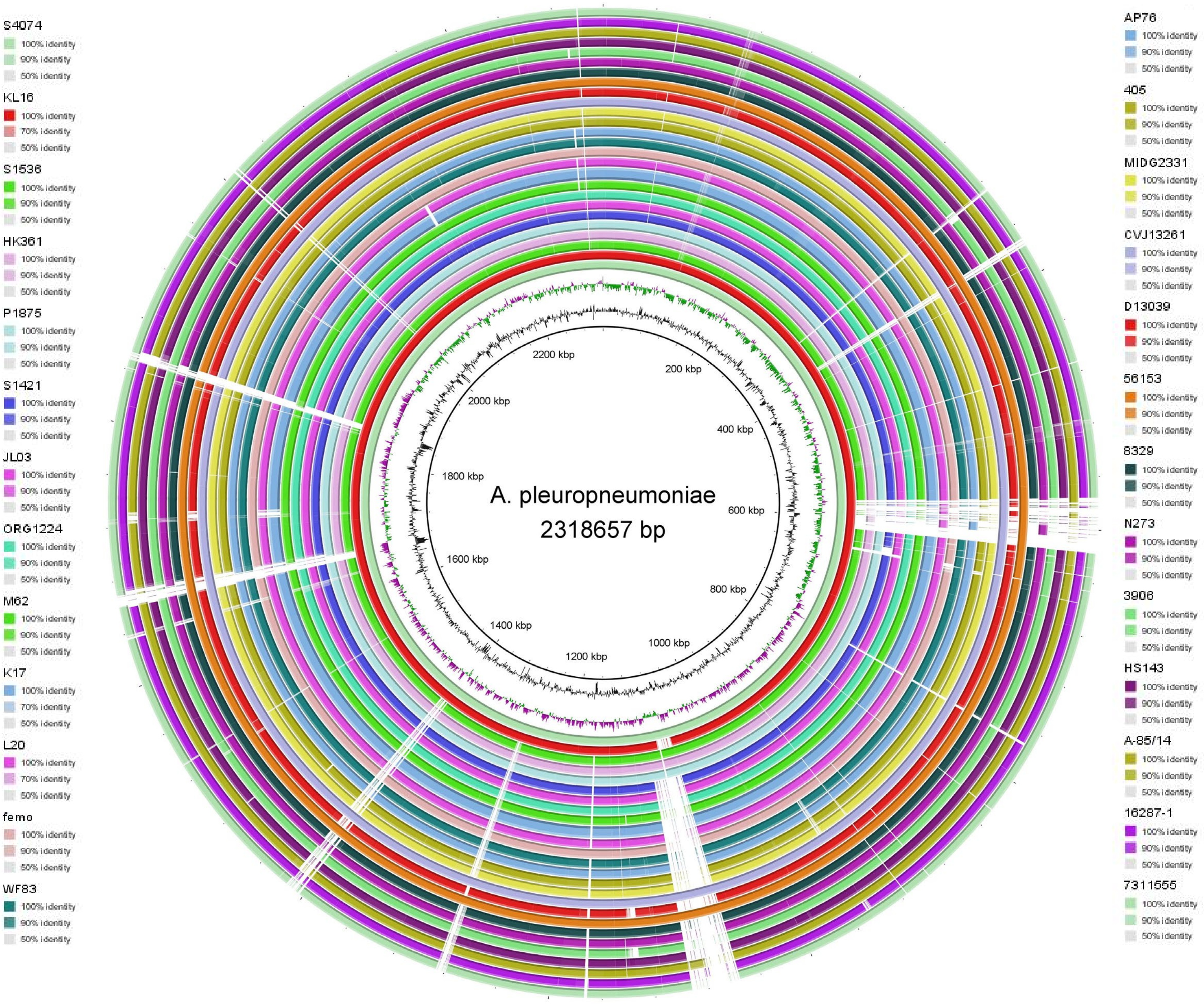

Figure S2. Circular maps depicting whole-genome comparisons of the 26 *A. pleuropneumoniae* strains. The scale ring shows the coordinates of the serotype 1 reference strain S4074 in kilobase pairs. The second ring represents the average GC content. The third ring represents the GC skew. The colored outer rings display regions of homology among the 26 *A. pleuropneumoniae* strains based on BLASTn alignments.

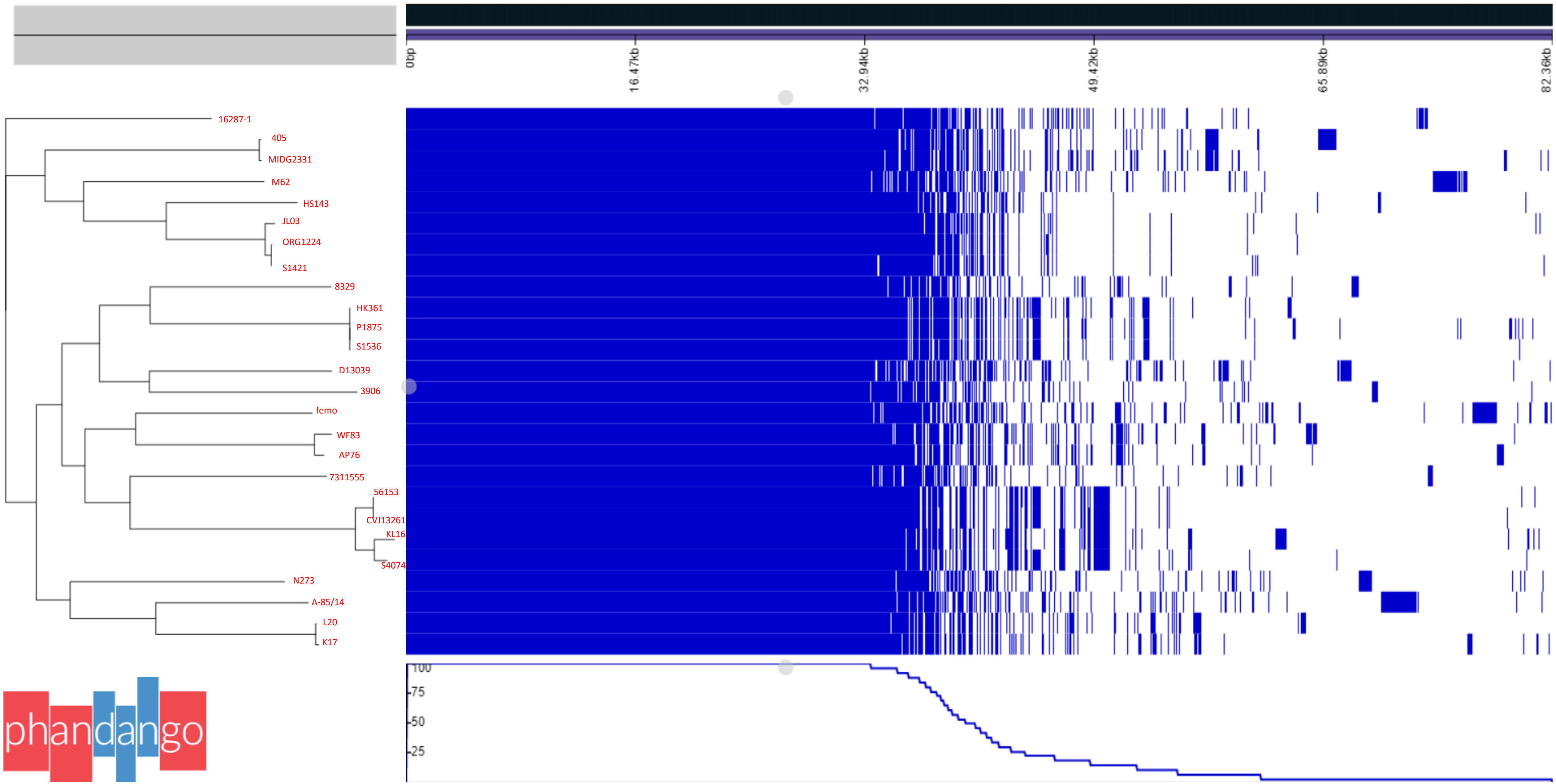

Figure S3. Gene distribution of the pangenome of the 26 *A. pleuropneumoniae* strains. On the left side of the figure is an unrooted tree inferred with FastTree from the MAFFT-based core-genome alignment computed with Roary. In the centre, the distribution of the 4,116 genes of the pangenome are displayed, with blue segments representing gene presence and white segments representing gene absence. The pangenome is displayed, starting from the core genome on the left and transitioning into the accessory genome (shell and cloud genomes) with increasing gene sequence disparity. The bottom graph displays a trace showing the percentage of isolates containing the same genes.

	KL16 serotype ...	HK361 serotype ...	P1875 serotype ...	JL03 serotype ...	ORG1224 serotype ...	AP76 serotype ...	MIDG2331 serotype ...	S4074 serotype ...	D13039 serotype ...	56153 serotype ...	8329 seotype ...	N273 serotype ...	3906 serotype ...	HS143 serotype ...	A-85/14 serotype ...	16287-1 serotype ...	7311555 serotype ...	S1536 serotype ...	S1421 serotype ...	M62 serotype ...	K17 serotype ...	L20 serotype ...	femo serotype ...	WF83 serotype ...	405 serotype ...	CVJ13261 serotype ...
serotype 1 KL16		97.072%	97.008%	96.755%	97.255%	97.090%	97.431%	98.530%	97.060%	99.155%	97.514%	96.948%	97.244%	97.382%	96.853%	97.457%	97.255%	97.034%	97.437%	97.247%	96.749%	96.887%	97.213%	97.756%	97.327%	99.184%
serotype 2 HK361	97.072%		99.850%	97.596%	97.779%	97.437%	97.280%	97.415%	97.584%	97.435%	97.868%	97.604%	97.667%	97.543%	97.677%	97.523%	97.409%	99.890%	97.627%	97.437%	97.517%	97.105%	97.847%	97.389%	97.361%	97.476%
serotype 2 P1875	97.008%	99.850%		97.533%	97.717%	97.371%	97.217%	97.347%	97.519%	97.370%	97.805%	97.540%	97.603%	97.480%	97.611%	97.457%	97.346%	99.806%	97.567%	97.373%	97.451%	97.039%	97.784%	97.325%	97.297%	97.404%
serotype 3 JL03	96.755%	97.596%	97.533%		99.329%	97.345%	97.136%	97.317%	97.428%	97.157%	97.348%	97.484%	97.269%	97.930%	97.514%	97.199%	97.084%	97.559%	99.152%	97.449%	97.375%	96.761%	97.675%	97.164%	97.205%	97.190%
serotype 3 ORG1224	97.255%	97.779%	97.717%	99.329%		97.364%	97.710%	97.407%	97.515%	97.508%	97.925%	97.504%	97.777%	98.508%	97.594%	97.701%	97.566%	97.743%	99.787%	97.935%	97.451%	97.324%	97.675%	97.731%	97.779%	97.540%
serotype 7 AP76	97.090%	97.437%	97.371%	97.345%	97.364%		97.019%	97.195%	97.583%	97.523%	97.108%	97.531%	97.119%	97.058%	97.407%	97.303%	97.190%	97.408%	97.195%	96.877%	97.168%	96.516%	97.786%	98.877%	97.101%	97.531%
serotype 8 MIDG2331	97.431%	97.280%	97.217%	97.136%	97.710%	97.019%		96.834%	97.280%	97.237%	97.721%	97.223%	97.537%	97.761%	97.162%	97.893%	97.575%	97.237%	97.888%	97.709%	97.004%	97.231%	97.140%	97.928%	99.674%	97.272%
serotype 1 S4074	98.530%	97.415%	97.347%	97.317%	97.407%	97.195%	96.834%		97.668%	98.812%	97.354%	97.170%	97.027%	97.102%	97.595%	96.837%	96.830%	97.373%	97.249%	97.054%	97.544%	96.896%	97.771%	96.993%	96.897%	98.847%
serotype 10 D13039	97.060%	97.584%	97.519%	97.428%	97.515%	97.583%	97.280%	97.668%		97.387%	97.502%	97.551%	97.380%	97.156%	97.707%	97.223%	97.225%	97.541%	97.358%	97.046%	97.630%	96.968%	97.774%	97.443%	97.338%	97.423%
serotype 11 56153	99.155%	97.435%	97.370%	97.157%	97.508%	97.523%	97.237%	98.812%	97.387%		97.433%	97.400%	97.142%	97.140%	97.254%	97.241%	97.184%	97.398%	97.355%	97.098%	97.093%	96.741%	97.491%	97.588%	97.324%	99.953%
seotype 12 8329	97.514%	97.868%	97.805%	97.348%	97.925%	97.108%	97.721%	97.354%	97.502%	97.433%		97.294%	98.075%	97.937%	97.427%	97.738%	97.771%	97.825%	98.105%	97.804%	97.195%	97.399%	97.439%	97.846%	97.630%	97.467%
serotype 13 N273	96.948%	97.604%	97.540%	97.484%	97.504%	97.531%	97.223%	97.170%	97.551%	97.400%	97.294%		97.300%	97.090%	97.389%	97.435%	97.425%	97.565%	97.347%	97.149%	97.236%	96.693%	97.476%	97.296%	97.296%	97.429%
serotype 14 3906	97.244%	97.667%	97.603%	97.269%	97.777%	97.119%	97.537%	97.027%	97.380%	97.142%	98.075%	97.300%		97.700%	97.291%	97.618%	97.638%	97.629%	97.948%	97.707%	97.083%	97.283%	97.348%	97.760%	97.445%	97.171%
serotype 15 HS143	97.382%	97.543%	97.480%	97.930%	98.508%	97.058%	97.761%	97.102%	97.156%	97.140%	97.937%	97.090%	97.700%		97.298%	97.718%	97.574%	97.505%	98.700%	97.935%	97.194%	97.410%	97.489%	97.732%	97.549%	97.168%
serotype 16 A-85/14	96.853%	97.677%	97.611%	97.514%	97.594%	97.407%	97.162%	97.595%	97.707%	97.254%	97.427%	97.389%	97.291%	97.298%		97.292%	97.082%	97.633%	97.427%	97.175%	98.418%	97.667%	97.879%	97.208%	97.240%	97.290%
serotype 17 16287-1	97.457%	97.523%	97.457%	97.199%	97.701%	97.303%	97.893%	96.837%	97.223%	97.241%	97.738%	97.435%	97.618%	97.718%	97.292%		97.815%	97.479%	97.878%	97.826%	97.138%	97.355%	97.255%	98.022%	97.814%	97.277%
serotype 18 7311555	97.255%	97.409%	97.346%	97.084%	97.566%	97.190%	97.575%	96.830%	97.225%	97.184%	97.771%	97.425%	97.638%	97.574%	97.082%	97.815%		97.365%	97.744%	97.511%	97.000%	97.227%	97.195%	97.791%	97.490%	97.221%
serotype 2 S1536	97.034%	99.890%	99.806%	97.559%	97.743%	97.408%	97.237%	97.373%	97.541%	97.398%	97.825%	97.565%	97.629%	97.505%	97.633%	97.479%	97.365%		97.589%	97.394%	97.474%	97.063%	97.817%	97.360%	97.319%	97.429%
serotype 3 S1421	97.437%	97.627%	97.567%	99.152%	99.787%	97.195%	97.888%	97.249%	97.358%	97.355%	98.105%	97.347%	97.948%	98.700%	97.427%	97.878%	97.744%	97.589%		98.119%	97.280%	97.499%	97.516%	97.910%	97.787%	97.384%
serotype 4 M62	97.247%	97.437%	97.373%	97.449%	97.935%	96.877%	97.709%	97.054%	97.046%	97.098%	97.804%	97.149%	97.707%	97.935%	97.175%	97.826%	97.511%	97.394%	98.119%		97.008%	97.216%	97.275%	97.684%	97.641%	97.134%
serotype 5a K17	96.749%	97.517%	97.451%	97.375%	97.451%	97.168%	97.004%	97.544%	97.630%	97.093%	97.195%	97.236%	97.083%	97.194%	98.418%	97.138%	97.000%	97.474%	97.280%	97.008%		99.138%	97.849%	96.975%	97.088%	97.130%
serotype 5b L20	96.887%	97.105%	97.039%	96.761%	97.324%	96.516%	97.231%	96.896%	96.968%	96.741%	97.399%	96.693%	97.283%	97.410%	97.667%	97.355%	97.227%	97.063%	97.499%	97.216%	99.138%		97.112%	97.195%	97.163%	96.778%
serotype 6 femo	97.213%	97.847%	97.784%	97.675%	97.675%	97.786%	97.140%	97.771%	97.774%	97.491%	97.439%	97.476%	97.348%	97.489%	97.879%	97.255%	97.195%	97.817%	97.516%	97.275%	97.849%	97.112%		97.550%	97.200%	97.520%
serotype 7 WF83	97.756%	97.389%	97.325%	97.164%	97.731%	98.877%	97.928%	96.993%	97.443%	97.588%	97.846%	97.296%	97.760%	97.732%	97.208%	98.022%	97.791%	97.360%	97.910%	97.684%	96.975%	97.195%	97.550%		97.834%	97.618%
serotype 8 405	97.327%	97.361%	97.297%	97.205%	97.779%	97.101%	99.674%	96.897%	97.338%	97.324%	97.630%	97.296%	97.445%	97.549%	97.240%	97.814%	97.490%	97.319%	97.787%	97.641%	97.088%	97.163%	97.200%	97.834%		97.362%
serotype 9 CVJ13261	99.184%	97.476%	97.404%	97.190%	97.540%	97.531%	97.272%	98.847%	97.423%	99.953%	97.467%	97.429%	97.171%	97.168%	97.290%	97.277%	97.221%	97.429%	97.384%	97.134%	97.130%	96.778%	97.520%	97.618%	97.362%	

Figure S4. Core-genome similarity matrix of the 26 *A. pleuropneumoniae* strains. Values (% identity) were obtained by sequence alignment of the core-genomes computed with Roary.

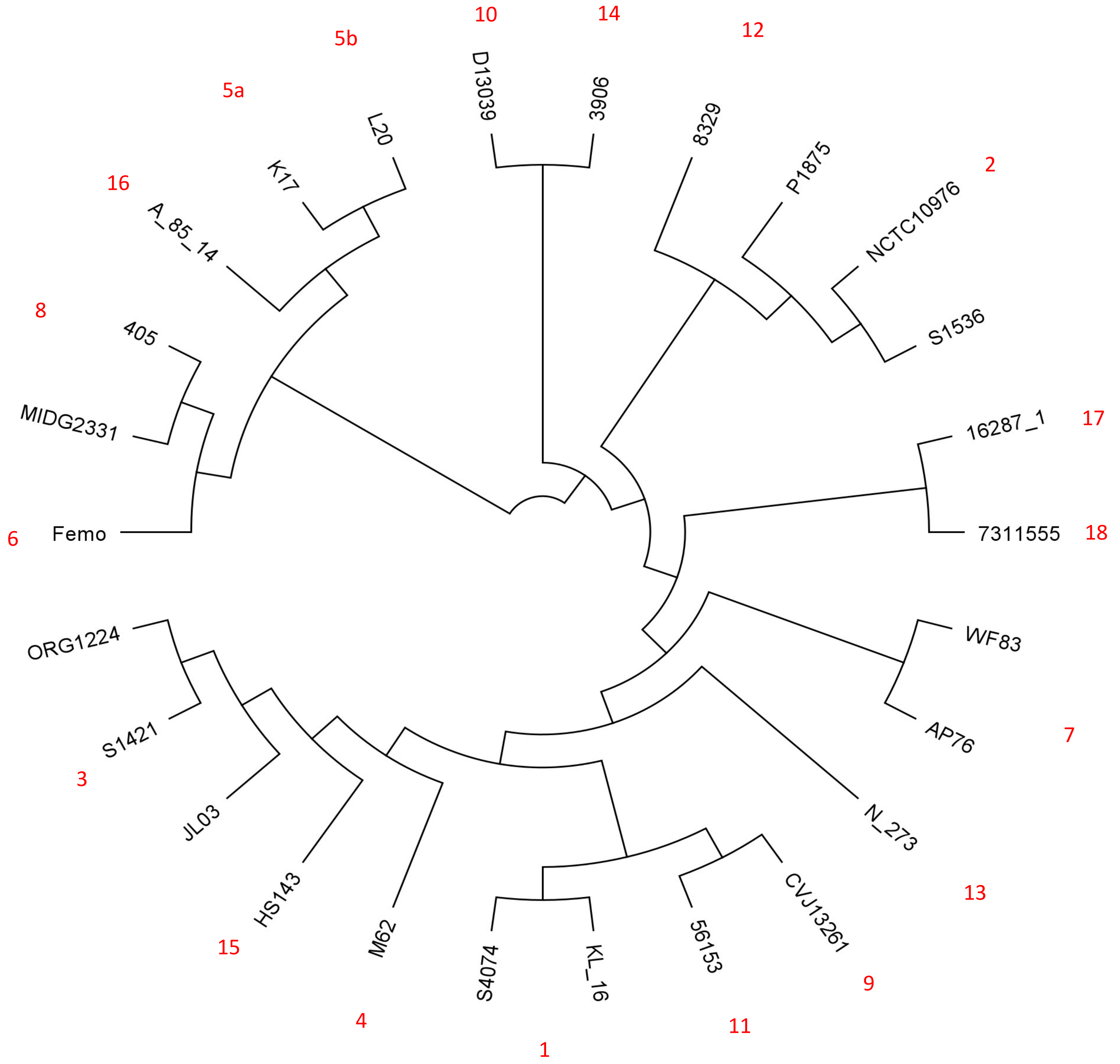

Figure S5. Phylogeny of the 26 *A. pleuropneumoniae* strains based on core-genome multilocus sequence typing (cgMLST). Identification of the MLST targets and tree generation were performed with SeqSphere. Serotype numbers are highlighted in red.
